# Termite’s Twisted Mandible Presents Fast, Powerful, and Precise Strikes

**DOI:** 10.1038/s41598-020-66294-1

**Published:** 2020-06-11

**Authors:** Kuan-Chih Kuan, Chun-I Chiu, Ming-Chih Shih, Kai-Jung Chi, Hou-Feng Li

**Affiliations:** 1 Department of Entomology, National Chung Hsing University, Taichung City, Taiwan; 2 Department of Physics and Institute of Biophysics, National Chung Hsing University, Taichung City, Taiwan; 3 Department of Life Sciences, National Chung Hsing University, Taichung City, Taiwan; 4 The iEGG and Animal Biotechnology Center, National Chung Hsing University, Taichung City, Taiwan

**Keywords:** Animal behaviour, Biomechanics, Entomology

## Abstract

The asymmetric mandibles of termites are hypothetically more efficient, rapid, and powerful than the symmetric mandibles of snap-jaw ants or termites. We investigated the velocity, force, precision, and defensive performance of the asymmetric mandibular snaps of a termite species, *Pericapritermes nitobei*. Ultrahigh-speed recordings of termites revealed a new record in biological movement, with a peak linear velocity of 89.7–132.4 m/s within 8.68 μs after snapping, which caused an impact force of 105.8–156.2 mN. High-speed video recordings of ball-strike experiments on termites were analysed using the principle of energy conservation; the left mandibles precisely hit metal balls at the left-to-front side with a maximum linear velocity of 80.3 ± 15.9 m/s (44.0–107.7 m/s) and an impact force of 94.7 ± 18.8 mN (51.9–127.1 mN). In experimental fights between termites and ant predators, *Pe. nitobei* killed 90–100% of the generalist ants with a single snap and was less likely to harm specialist ponerine ants. Compared with other forms, the asymmetric snapping mandibles of *Pe. nitobei* required less elastic energy to achieve high velocity. Moreover, the ability of *P. nitobei* to strike its target at the front side is advantageous for defence in tunnels.

## Introduction

Some invertebrates’ elastic power-amplifying systems that incorporate latches and springs can overcome the physiological limits of muscle contraction and perform a powerful or rapid movement^1,2^. For example, mantis shrimps (Crustacea: Stomatopoda) perform high-speed strikes to prey by using elastic energy stored in their raptorial appendages^3^. Springtails (Hexapoda: Collembola) escape from enemies with quick and powerful jumps by using their springing organ^4^. Many ants and termites, such as the snap-jaw ant *Mystrium camillae* Emery (Hymenoptera: Formicidae)^5^ and the soldiers of termite *Termes panamaensis* (Snyder) (Blattodea: Termitidae)^6^, have mandibles that are morphologically specialised for powerful snapping attacks. The snapping speeds of the mandibular attacks of *M*. *camillae* (111.1 m/s)^5^ and *T*. *panamaensis* (67 m/s)^6^ are the most rapid animal movements currently reported^1,5,6^, followed by the mandible-closing movement of *Odontomachus bauri* Emery trap-jaw ants (64.3 m/s)^7^, the diving of gyrfalcons (58 m/s)^8^, the nematocyst discharge of jellyfish (37 m/s)^9^, and strike behaviour of mantis shrimps (31 m/s)^1^.

**Figure 1:**
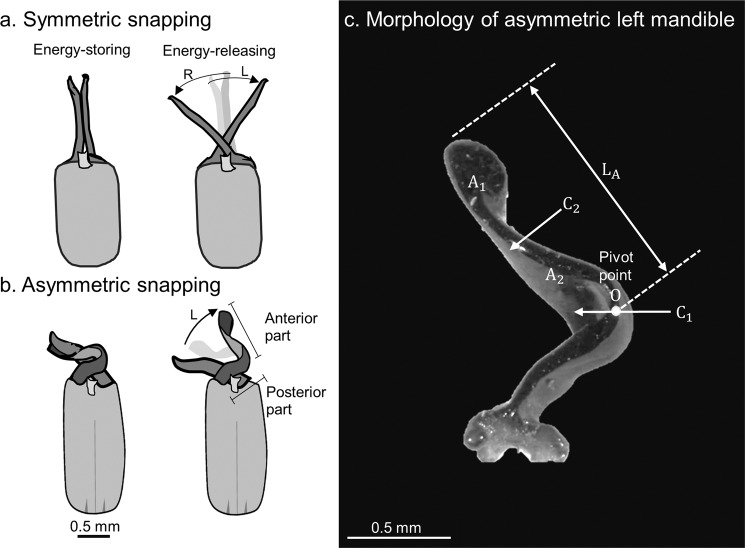
Snapping mandibles of termite soldiers. (**a**) In the symmetric snapping of *Termes panamaensi*^6,10^, elastic energy is stored in both mandibles, as indicated by the deformation and motion of the left (L) and right (R) mandibles. (**b**) In the asymmetric snapping of *Pericapritermes* spp.^10^, elastic energy is stored in only the left mandible when its anterior part rotates about the joint or pivot point O. The posterior part remains stationary during snapping. (**c**) Morphology of the twisted left mandible of the *Pericapritermes nitobei* termite used in this study. The length of the anterior part of the left mandible (*L*_A_) and the mass of its subsections A_1_ and A_2_ (i.e., *M*_A1_, *M*_A2_) are required to estimate its moment of inertia (*I*_A_). The subsections A_1_ and A_2_ were prepared by performing two cuts; one cut was made at the pivot point (C_1_) and the other was made at the centre of the anterior part (C_2_).

The snapping mandibles of termite soldiers have two forms: symmetric and asymmetric^10,11^. *T. panamaensis* has two narrow and elongated symmetric snapping mandibles that press together until they slide against each other (Fig. 1a). These mandibles strike enemies along their lateral sides^6^. By contrast, the asymmetric snapping mandibles of some termites are relatively short and wide, with a twisted left mandible and curved right mandible^10^. The right mandible presses against the left until the two surfaces slide. Moreover, the left mandible snaps in a clockwise motion (Fig. 1b), presumably striking enemies along the front side. Additionally, asymmetric snapping mandibles may perform more violent strikes than symmetric mandibles can by storing elastic energy in the left twisted mandible^12^. However, no conclusive evidence has supported these hypotheses.

Ants are major natural predators of termites. Predator ants include specialist predators such as *Pachycondyla* spp. (Formicidae: Ponerinae)^13^ and generalist predators such as *Anoplolepis* spp. and *Pheidole* spp.^14,15^. The powerful mandibular snapping of termite soldiers is hypothesised to be a specialised defence mechanism against ants^16^. Termites can snap the ground to leap away from ants^17,18^ or snap at ants to push them away^16^ or even kill them^6^. However, these defensive behaviours have not been studied in termites with asymmetric mandibles.

This study investigated the velocity, force, precision, and defensive behaviours of asymmetric mandibular snaps performed by *Pericapritermes nitobei* (Shiraki) soldiers. Specifically, we conducted ultrahigh- and high-speed video recordings, ball-strike experiments, and ant-defence assessments to test the following hypotheses: (1) snaps of asymmetric mandibles are more rapid and powerful than snaps of symmetric mandibles are, (2) asymmetric mandibles can precisely strike at the front side, and (3) asymmetric mandibles are effective for defending against ants. In addition to revealing the fastest known animal movement, we compared asymmetric and symmetric termite mandibles and analysed the evolution and mechanical effects of mandible morphology.

## Methods

### Termites and ants

A total of 100 *Pe. nitobei* termite soldiers were collected by excavating the soil beneath stones and logs in three locations in Taiwan, namely Xiaping Tropical Botanical Garden (23.77°N, 120.67°E), Huisun Forest Station (24.09°N, 121.03°E), and Dakeng (24.19°N, 120.79°E) (Supplementary Information, Table S1). *Pe. nitobei* is a soil-feeding termite that does not have a centralised nest; they distribute their eggs and larvae in subterranean tunnels beneath stones^19^. They generally forage on organic matter adjacent to plant roots and rarely appear on the ground surface^19^. Because *Pe. nitobei* colonies are difficult to maintain in laboratory conditions, all experiments were conducted within 1 day after collection. The sample sizes and collection sites of subjects used in all the experiments are summarised in Table S1 of the Supplementary Information.

Natural predators of *Pe. nitobei* were identified on the basis of references and field observations. *Pheidole* and *Anoplolepis* spp. are generalist termite predators^14,15^, and *Anochetus* and *Pachycondyla* spp. are specialist predators of termites that perform specialised striking behaviours on termite soldiers or recognise termite odours and raid termite nests^20,21^. In this study, *Ph. megacephala* (Formicidae: Myrmecinae) and *Anop. gracilipes* (Formicidae: Formicinae) were assumed to be generalist predators of *Pe. nitobei*. Both species have been observed to forage on the ground and prey on termites in Xiaping Tropical Botanical Garden^22,23^. Two ponerine ant species (Formicidae: Ponerinae), namely *Anochetus taiwaniensis* and *Pa. javanus*, were assumed to be specialist predators of *Pe. nitobei*. *Anoc. taiwaniensis* was observed foraging for termites in rotting logs at Huisun Forest Station. *Pa. javanus* was observed foraging in the soil surrounding the subterranean galleries of *Pe. nitobei* in Xiaping Tropical Botanical Garden, Huisun Forest Station, and Dakeng. The generalist and specialist ants used in the assessments were collected from National Chung Hsing University and Huisun Forest Station, respectively (Supplementary Information, Table S1).

### Estimating velocity and force by using an ultrahigh-speed camera

In each experiment, a termite was placed in the centre of a plastic petri dish (diameter: 90 mm) with a filter paper (Advantec No. 1, Toyo Inc.; diameter: 90 mm). Snapping behaviour was then recorded at 460,830 frames per second (fps) by using an ultrahigh-speed video camera (Model: v2512, Phantom, New Jersey, USA) with a resolution of 128 × 128 pixels^2^. The linear velocity of the mandible tip (*V*_MT_) was calculated as follows:

1 $${V}_{MT}={L}_{A}{\omega }_{A},$$

where *L*_A_ and *ω*_A_ are the length and angular velocity of the anterior rotating part about the pivot point O (Fig. 1c), respectively. The parameter *ω*_A_ was calculated from the video footage by using Phantom CV 2.8 (Phantom, New Jersey, USA) for image analysis.

The snapping force (*F*_A_) was calculated using the following equation:^6,7^

2 $${F}_{A}=\frac{1}{3}{M}_{A}{L}_{A}{\alpha }_{A},$$

where *M*_A_ and *α*_A_ are the mass and angular acceleration of the anterior rotating part of the left mandible, respectively. The average *α*_A_ can be calculated as follows:

3 $${\alpha }_{A}=\frac{{\omega }_{A}}{{t}_{A}},$$

where *t*_A_ is the time required to reach the maximum *ω*_A_. The snapping energy (*E*_A_) (i.e., the kinetic energy due to rotation) was calculated as follows:

4 $${E}_{A}=\frac{1}{2}{I}_{A}{\omega }_{A}^{2}$$

where *I*_A_ is the moment of inertia of the anterior rotating part about the pivot point O (Fig. 1c). Because the termite’s left mandible cannot be modelled using a common geometrical form, we adjusted the moment of inertia (*I*_A_) by using a constant *K* as follows:

5 $${I}_{A}=K{M}_{A}{L}_{A}^{2}.$$

The parameter *K* was calculated using the location of the anterior left mandible’s centre of mass (*L*_COM_), length of the anterior mandible part (*L*_A_), and mass of mandible parts A_1_ (*M*_A1_) and A_2_ (*M*_A2_) (Fig. 1c; Table S3) as follows:

6 $$K=\frac{{M}_{A2}}{{M}_{A}}\cdot {\left(\frac{{L}_{COM}}{2{L}_{A}}\right)}^{2}+\frac{{M}_{A1}}{{M}_{A}}\cdot {\left(\frac{{L}_{COM}+{L}_{A}}{2{L}_{A}}\right)}^{2},$$

and *L*_COM_ was calculated as follows:

7 $${L}_{COM}=\frac{1/4{L}_{A}{M}_{A2}+3/4{L}_{A}{M}_{A1}}{{M}_{A}}.$$

The termite’s left mandible is morphologically inhomogeneous. The left mandible is wide in the posterior part and at the pivot point and narrow at the centre of the anterior part (Fig. 1c). To obtain parts A_1_ and A_2_, we used a scalpel blade to cut the left mandible first at its pivot point (C_1_) and then in the middle of the anterior part (C_2_) (Fig. 1c). To avoid measurement errors, *M*_A1_ and *M*_A2_ were calculated as the means of 12 A_1_ and 14 A_2_ parts, respectively. The total mass of the anterior left mandible (*M*_A_) was the sum of *M*_A1_ and *M*_A2_.

A total of 17 soldiers were used to measure the mass and length of the left mandible (Table S1). Images were captured using a Leica M205 C stereomicroscope with a Leica MC170 HD digital camera (Leica Microsystems, Wetzlar, Germany), and all lengths were calculated using the LAS Image Analysis software module (Leica Application Suite V4.4.0, Leica Microsystems, Wetzlar, Germany). The mass of the termites was measured using an analytical balance (AG245, Mettler Toledo, Greifensee, Switzerland).

Termite behaviours were considered abnormal in ultrahigh-speed recordings under the following conditions: (1) three of five termites died in the petri dish before snapping, (2) the other two termites snapped without being triggered and did not extend their antennae forward to detect targets in front of them, and (3) the termites died immediately after snapping once. We assumed that the bright light required for ultra-speed recording (brightness of approximately 85,000 lux) caused these abnormal behaviours. Therefore, we used a high-speed video camera with less intense light (approximately 8,670 lux) to observe the snapping behaviour and reassess the snapping velocity and force. To minimise the effects of light on ant and termite behaviour, we recorded fights between termites and ants by using a smartphone camera with a light intensity of approximately 850 lux.

### Estimating mandible strike accuracy, velocity, and force by using a high-speed camera

Each termite was placed in a plastic petri dish with a filter paper, as in the ultrahigh-speed recordings. Mandible-snapping behaviour was triggered by touching a termite’s antennae from the front by using tweezers. Snapping was recorded at 1,000 fps with a high-speed video camera (MotionPro X3, Integrated Design Tools, Inc., Tallahassee, FL, USA). Behavioural phases were identified, and the duration of each phase was measured using the recorded images.

To measure the striking angle and reassess the velocity and force of mandible snapping, we conducted ball-strike experiments on 15 soldiers. In each experiment, a metal ball (2.25 mg, 0.7 mm in diameter) with a mass similar to that of the termite was obtained from the tip of a ballpoint pen (TOWO OP-100, 0.7 mm) and placed in front of the soldier’s head. The soldiers snapped at the ball 1–2 seconds after it was placed. When the left mandible hit the metal ball, the termite soldier and the ball moved away from each other; movements were recorded by the aforementioned high-speed video camera at 1,000 fps. The diameter of the petri dish was used as a scale for distance measurement. The experiment was repeated until the termite was exhausted and unresponsive to stimuli. For each termite, 3–14 snaps were recorded.

Assuming that no energy loss caused by air resistance or ball rotation occurred, according to the energy conservation principle, the kinetic energy of the rotating anterior part of the left mandible (*E*_A_) on striking the ball equals the sum of the linear and angular kinetic energy of the ball and termite as follows:

8 $${E}_{A}=\frac{1}{2}{I}_{A}{\omega }_{A}^{2}=\frac{1}{2}{M}_{B}{V}_{B}^{2}+\frac{1}{2}{M}_{T}{V}_{T}^{2}+\frac{1}{2}{I}_{T}{\omega }_{T}^{2},$$

where *M*_B_ and *M*_T_ are the masses of the metal ball (2.25 mg) and termite, respectively; *V*_B_ and *V*_T_ are the linear velocities of the ball and termite, respectively; *ω*_T_ is the angular velocity of the termite; and *I*_T_ is the moment of inertia of the termite. The parameters *V*_B_, *V*_T_, and *ω*_T_ were measured according to the motions of the termite and ball by using video analysis software (Tracker 4.91, http://physlets.org/tracker/). The parameters *V*_T_ and *V*_B_ were calculated from the linear displacement of the termite and ball between the first two frames after the termite struck the ball. The variable *ω*_T_ was calculated using the termite’s number of rotations over a specific time period. By assuming the termite to resemble a cylinder rotating about its centre of mass, its *I*_T_ was calculated from its mass (*M*_T_) and body length (*L*_T_) as follows:^24^

9 $${I}_{T}=\frac{1}{12}{M}_{T}{L}_{T}^{2}.$$

Subsequently, the snapping force (*F*_A_) could be calculated using Eqs. (2) and (3). The linear velocity of the tip of the snapping left mandible on striking the ball (*V*_MT_) could be calculated using Eqs. (1), (4), and (5) as follows:

10 $${V}_{MT}={L}_{A}{\omega }_{A}=\sqrt{\frac{2{E}_{A}}{{M}_{A}K}},$$

where *K* is a constant for adjusting the moment of inertia (*I*_A_) and *M*_A_ is the total mass of the anterior left mandible. The parameter *K* was calculated using Eqs. 6 and 7, and *E*_A_ was calculated using Eq. 8.

We evaluated the precision of mandibular snaps by variations in the ball’s movement directions after a snap. Precise snaps occurred in a particular hitting zone with less variation in movement directions. The ball’s movement directions were calculated as the angle of deviation from the termite’s body axis. To determine whether the snaps had a particular hitting zone, we performed a Shapiro–Wilk test for confirming the normality of angle measurements.

**Figure 2:**
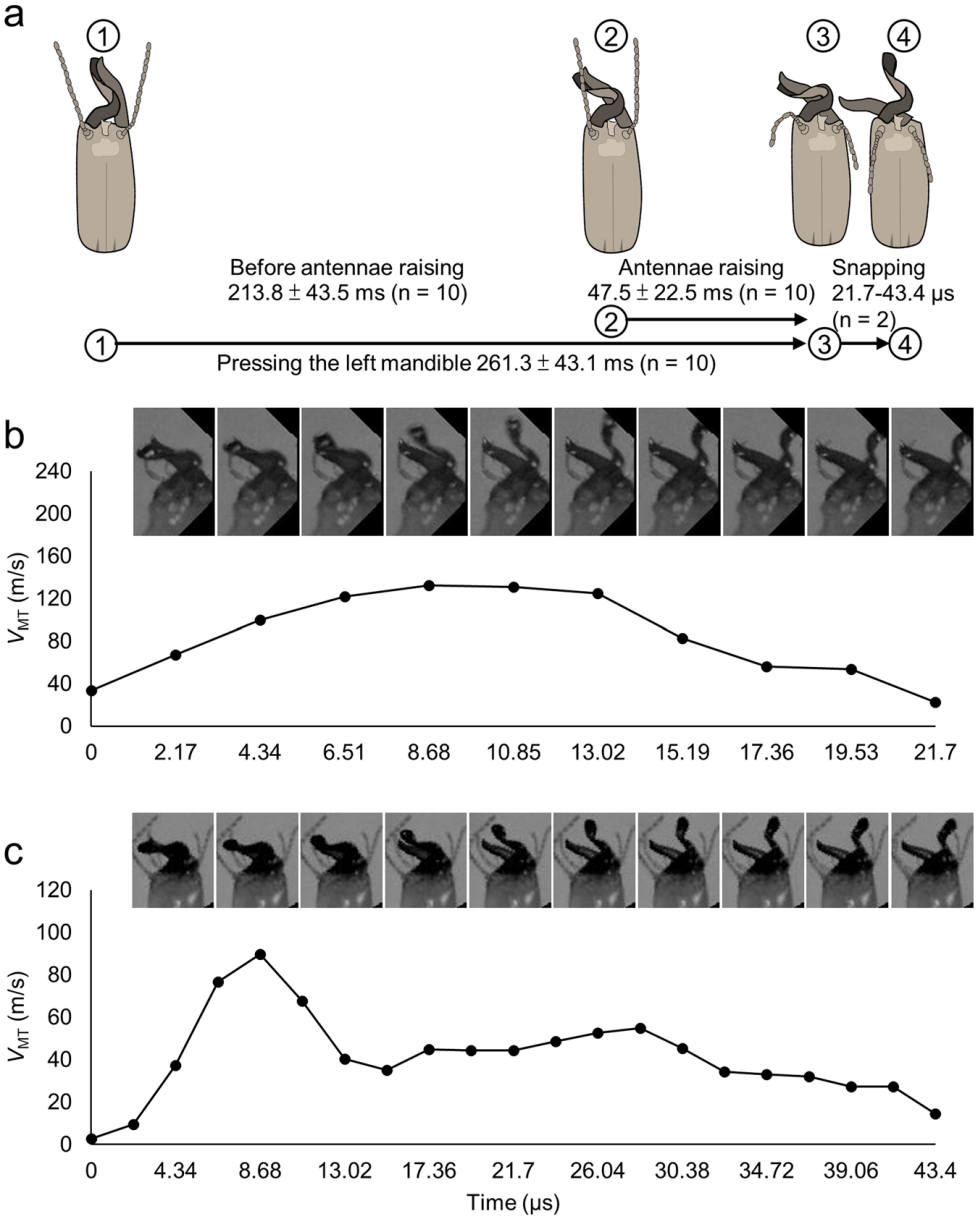
Behavioural phases of *Pericapritermes nitobei* mandibular snaps. (**a**) Stages 1–3: High-speed recordings from 10 soldiers at 1,000 frames per second (fps) indicated that the right mandible pressed against the left mandible for 261 ± 43 ms before the mandibles slid across each other to snap, and termites raised their antennae 47.5 ± 22.5 ms before the snap (Stages 2–3). Stage 3–4: Single snap of two soldiers (b and c) recorded by an ultrahigh-speed video camera at 460,830 fps. Mandibular snaps were performed over 21.7–43.4 μs. The peak linear velocity (*V*_MT_) of two soldiers at 8.68 μs were 132.4 and 89.7 m/s. Images in (**b**,**c**) were obtained by transforming Supplementary Movies S1 and S2, respectively, to frames using codes written in Python language (v. 3.8) with the package *opencv-python*.

**Figure 3:**
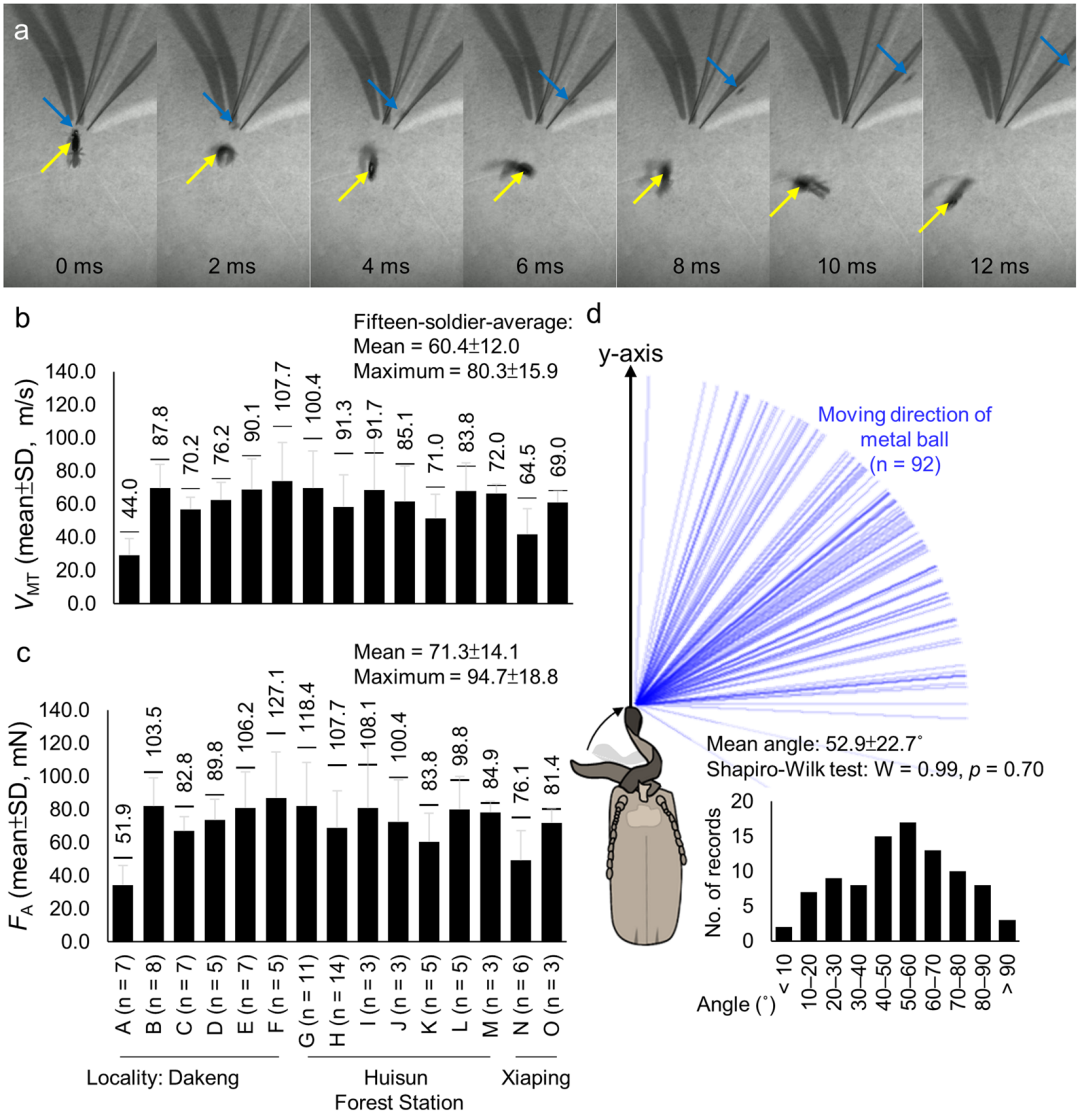
Snap performance of the *Pericapritermes nitobei* soldier’s asymmetric mandible in ball-strike experiments (92 events from 15 termites, A–O). (**a**) Motion of the termites and metal ball. When the left mandible hit the metal ball, the termite soldier and ball moved away from each other. Yellow and blue arrows indicate the position of the termite and metal ball, respectively. (**b**) Linear velocity *V*_MT_. (**c**) Snapping force *F*_A_. The maximum recorded *V*_MT_ and *F*_A_ values for each soldier are displayed above each bar. (**d**) Movement directions of the metal ball. Each blue line indicates a record of one moving metal ball. Angle normality was assessed using the Shapiro–Wilk test. Images in (**a**) were obtained by transforming Supplementary Movie S3 to frames using codes written in Python language (v. 3.8) with the package *opencv-python*.

### Assessment of termites defending against ants

To observe snapping behaviour against predators, termite soldiers were forced to fight with workers of *Anop. gracilipes*, *Anoc. taiwaniensis*, and *Pa. javanus* and minor workers of *Ph. megacephala*. The collection locations of ants and the number of termites used in each trial are presented in Table S1. Fights occurred in a petri dish (diameter: 55 mm) layered with a filter paper (Advantec No. 2, Toyo Inc.; diameter: 55 mm) containing 1 mL of water. In each trial, four termites and four ants of the same species were placed on the filter paper, and their fights were recorded at 30 fps for 15 minutes on a smartphone camera (ZenFone 3 ZE520KL, ASUSTeK Computer Inc.). We reconfirmed whether the ants were predators of *Pe. nitobei* according to the number of encounters with termites and the number of attacks performed by each ant species. To evaluate the termites’ defensive performance, the numbers of mandibular snaps and hits as well as ants killed were recorded. Six trials were performed for each ant species, and the data from all trials were combined to calculate the ants’ attack rates and termites’ hitting and killing probabilities. The attack rate of ants was the ratio of the number of attacks performed to the number of encounters with termites. For termites, the hitting probability was the ratio of the number of hits to the number of snaps and the killing probability was the ratio of the number of ants killed to the number of hits. Pairwise comparisons for hitting and killing probabilities were conducted using Fisher’s exact tests with Bonferroni corrections. All the statistical data were obtained using R software (v. 3.3.1, R Development Core Team, 2013)^25^.

## Results

### Mandible-snapping sequences

Figure 2a displays a behaviour schematic. In the high- and ultrahigh-speed recordings, the mandible-snapping behaviour of *Pe. nitobei* soldiers began with the right mandible pressed against the left mandible to store elastic energy (loading phase). The termites required 261.3 ± 43.1 ms (n = 10) to complete the loading phase: During the first 213.8 ± 43.5 ms, both antennae extended forward as the right mandible continued to press against the left mandible; it took another 47.5 ± 22.5 ms to turn their antennae towards their rear before snap. Mandibular sliding was completed in 21.7–43.4 μs (Fig. 2b,c).

### Velocity and force of mandible snaps

In the ultrahigh-speed recordings of mandible snapping, the peak linear velocity occurred at 8.68 μs (*t*_A_) after execution and had values of 132.4 (Fig. 2b; Supplementary Movie S1) and 89.7 m/s (Fig. 2c; Supplementary Movie S2) for two termites. The peak snapping energy of these termites was 41.4 and 19.0 μJ respectively, and their peak snapping forces were 156.2 and 105.8 mN, respectively.

In the ball-strike experiments, the energy from the termite’s mandible snap caused the termite and metal ball to move away from each other and sometimes rotate (Fig. 3a, Supplementary Movie S3). On the basis of size and velocity measurements of 15 termite soldiers in 92 events (Tables S2, S3, Supplementary Information), the snapping energy (*E*_A_) had a mean of 9.5 ± 3.2 μJ and maximum of 15.8 ± 5.9 μJ (range: 4.6–27.4 μJ). The mean and maximum linear velocities of the mandible tip on striking the ball (*V*_MT_) were 60.4 ± 12.0 and 80.3 ± 15.9 m/s (range: 44.0–107.7 m/s), respectively (Fig. 3b). Assuming that the left mandible struck the ball at its maximum linear velocity (*t*_A_) 8.68 μs after snapping (Fig. 2b,c), the mean and maximum snapping forces (*F*_A_) were 71.3 ± 14.1 and 94.7 ± 18.8 mN (range: 51.9–127.1 mN), respectively (Fig. 3c). The body measurement and snap performance data are summarised in Table 1.

**Table 1:** Comparison of body measurements and snap performance between the snapping termites and ants.

			Snapping termites			Trap-jaw ant	Snap-jaw ant	
			*Pericapritermes nitobei*^a^		*Termes panamaensi*^b^	*Odontomachus bauri*^c^	*Mystrium camillae*^d^	
							Major worker	Minor worker
Filming rate (kfps)			1	460	40	50	480	480
Body measurements	*M*_T_ (mg)	Min.	2.91		—	12.10	—	—
		Max.	3.39		—	14.90	—	—
		Mean	3.22		1.76	—	2.66	0.48
	*L*_T_ (mm)	Min.	5.90		—	—	—	—
		Max.	6.20		—	—	—	—
		Mean	6.06		5.00	—	—	—
	*M*_A_ (μg)	Min.	—		—	111.0	—	—
		Max.	—		—	145.0	—	—
		Mean	28.0		30.0	—	85.9	8.5
	*L*_A_ (mm)	Min.	0.97		—	1.24	—	—
		Max.	1.12		—	1.38	—	—
		Mean	1.06		1.50	—	1.56	0.84
	*I*_*A*_ (×10^–14^ kg·m^2^)	Min.	—		—	—	—	—
		Max.	—		—	—	—	—
		Mean	0.56		2.25	—	6.9	0.2
Snap Performance	*t*_A_ (μs)	Min.	—	8.68	—	—	—	—
		Max.	—	8.68	—	—	—	—
		Mean	—	8.68	25.0	130.0	34.8	14.6
	*V*_MT_ (m/s), peak	Min.	17.5	89.7	—	35.5	—	—
		Max.	107.7	132.4	67.0	64.3	107.4	111.1
		Mean	60.4	111.1	56.0	—	86.9	93.3
	*ω*_A_ (×10^4^ rad/s) peak	Min.	—	10.2	—	2.8	—	—
		Max.	—	15.3	—	4.7	—	—
		Mean	—	12.8	—	—	5.6	11.1
	*α*_A_ (×10^9^ rad/s^2^) peak	Min.	—	11.7	—	—	—	—
		Max.	—	17.6	—	—	—	—
		Mean	—	14.7	—	—	6.0	17.2
	*E*_A_ (μJ) peak	Min.	4.6	19.0	—	—	—	—
		Max.	27.4	41.4	—	—	—	—
		Mean	9.5	30.2	15.0	—	109.0	12.4
	*F*_A_ (mN) peak	Min.	20.4	105.8	—	—	—	—
		Max.	127.1	156.2	—	69	—	—
		Mean	71.3	131.0	54	47	268	41
	*F*_A_/body weight (×10^3^ BW)	Min.	1.6	3.9	—	0.4	—	—
		Max.	3.9	4.6	—	0.5	—	—
		Mean	3.0	4.2	3.1	—	10.3	8.7

Body measurements: *M*_T_, body mass; *L*_T_, body length; *M*_A_, mass of the waving part of left mandible; *L*_A_, length of the waving part of left mandible; *I*_A_, the moment of inertia of waving part of the left mandible. Snap performance: *t*_A_, duration of mandible strike; *V*_MT_, velocity of the mandible tip; *ω*_A_, angular velocity of waving part of the left mandible; *α*_A_, angular acceleration of mandible strike (calculated as *α*_A_ = *ω*_A_/*t*_A_); *E*_A_, energy released by mandible strike; *F*_A_, force caused by mandible acceleration; *F*_A_/body weight, ratio of maximum striking force and body weight.

^a^data from current study (see Tables S1, S2, and S3 in Electronic Supplementary Material);

^b^data from Seid *et al*.^6^;

^c^data from Patek *et al*.^7^;

^d^data from Larabee *et al*.^5^.

### Striking angle of mandible snaps

In the ball-strike experiments, the movement directions of the ball after being snapped by the *Pe. nitobei* mandible ranged from 2.3° to 122.1° and were normally distributed (Fig. 3d) (Shapiro–Wilk test: W = 0.99, *p* = 0.70) with a mean of 52.9° ± 22.7°. This result suggests that termites with asymmetric mandibles strike most enemies at the front-left side.

### Ant-defence performance

The generalist ants, namely *Ph. megacephala* and *Anop. gracilipes*, encountered termites 93 and 107 times and attacked termites 8 (8.6%) and 18 (17.1%) times in the defence experiments, respectively. The specialist ants, namely *Anoc. taiwaniensis* and *Pa. javanus*, encountered termites 300 and 143 times and attacked termites 102 (34.0%) and 33 (23.1%) times, respectively. The results indicated that *Ph. megacephala* and *Anop. gracilipes* are facultative or generalist predators of termites. Moreover, compared with *Ph. megacephala* and *Anop. gracilipes*, *Anoc. taiwaniensis* and *Pa. javanus* exhibited higher levels of aggression, which is consistent with them being specialist predators of termites.

**Figure 4:**
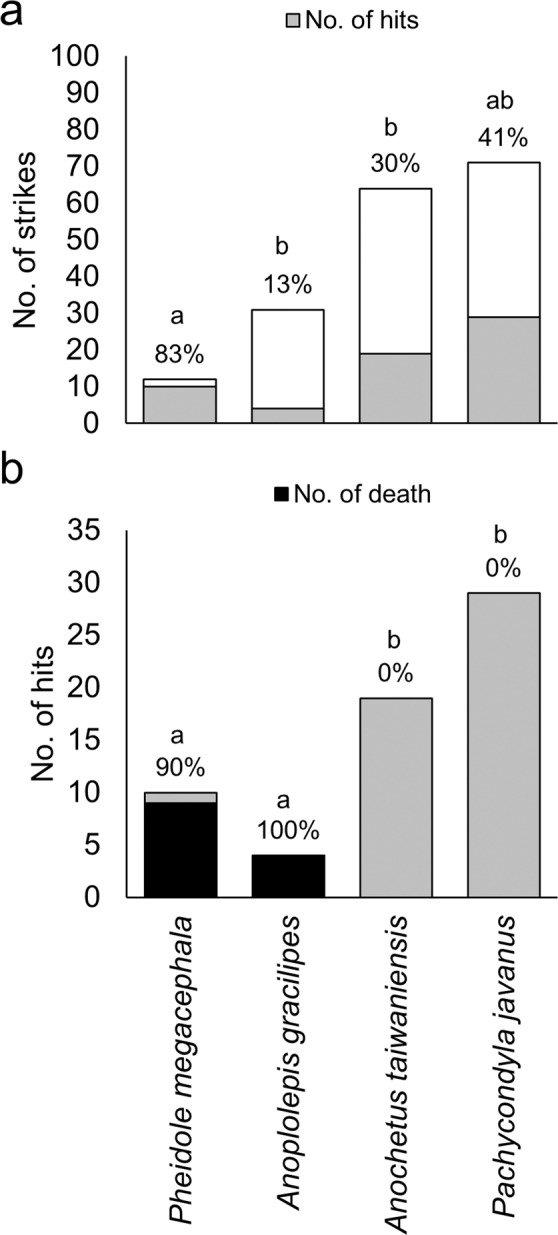
Defensive performance of mandibular snaps by probability (values above each bar) of (**a**) hitting and (**b**) killing four predator ant species. Bars with identical letters were not significantly different at *p* < 0.05 (Fisher’s exact tests with Bonferroni corrections). Termites could not kill their specialist predators *Anochetus taiwaniensis* and *Pachycondyla javanus*.

The termite soldiers had a significantly high probability of hitting *Ph. megacephala* (83%), significantly low probabilities of hitting *Anop. gracilipes* (13%) and *Anoc. taiwaniensis* (30%), and a moderate probability (41%) of hitting *Pa. javanus* (Fig. 4a) with a mandible snap. The mandibular snaps of termites killed most generalist ants in a single hit (mortality: 90–100%, Fig. 4b). By contrast, none of the ponerine ants were killed by a mandibular snap (Fig. 4b). Only 2 of 24 *Anoc. taiwaniensis* (8.3%) individuals were had immobilised left mandibles after being snapped by a termite.

## Discussion

### The most rapid animal movement

The most rapid recorded animal movement has been updated several times. The cheetah was considered to be the fastest animal before 1998^26^. The newest record was set by the snap-jaw ant *M. camillae*, whose mandible can achieve a maximum velocity of 93 ± 14.5 m/s^5^. Our results revealed that the snapping movement speed of *Pe. nitobei* soldiers’ asymmetric mandibles was similar to or even more rapid than that of snap-jaw ants’ symmetric mandible. Ultrahigh-speed recordings of snap-jaw ants indicated that 3 of 10 individuals had snapping velocities greater than 100.0 m/s, and the maximum recorded velocity was 111.1 m/s^5^. In our study of *Pe. nitobei*, the maximum snapping velocity calculated in the ball-strike experiment was 107.7 m/s, which was a conservative estimate because we disregarded the rotational energy of the metal ball and assumed no energy loss due to friction. The peak snapping velocity of *Pe. nitobei* calculated using ultrahigh-speed recordings reached 132.4 m/s, which was twice the velocity of the symmetric mandibles of *T. panamaensis* (67 m/s)^6^ and sets a new record for the most rapid biological movement.

### Powerful and efficient snapping mechanism of the asymmetric mandible

The ‘snap-jaw’ mechanism, which combines a spring and tool in one appendage, has been proposed to be more efficient than most other power-amplified appendages with distinct structures^2,5^. The peak impact force generated by a *Pe. nitobei* soldier may be approximately 4,600 times their body weight (BW), which is within the range estimated for other snap-jaw systems (3,000–10,000 BW). The aforementioned force is one order of magnitude higher than that reported for trap-jaw ants (approximately 500 BW)^7^ (Table 1).

Compared with symmetric mandibles, asymmetric mandibles require less energy to achieve super-rapid movement (Table 1). The average snapping velocity of *Pe. nitobei* soldiers calculated in the ball-strike experiment was similar to that reported for *T. panamaensis*^6^ (60.4 m/s vs. 56 m/s); however, the required elastic energy (*E*_A_) stored in the *Pe. nitobei* mandible was only two-thirds that stored in the *T. panamaensis* mandible (9.5 μJ vs. 15.0 μJ)^6^ even though *Pe. nitobei* has a body size twice that of *T. panamaensis*. At the peak snapping velocity of 132.4 m/s, *Pe. nitobei* soldiers must store 41.4 μJ of elastic energy, which is less than half of the 109 μJ of elastic energy required by major workers of *M. camillae* with similar body size and lower snapping velocity (111.1 m/s). Table 1 indicates that the lower energy requirement of *Pe. nitobei* asymmetric mandibles for achieving super-rapid movement can be attributed to their shorter rotating section (*L*_A_) and consequently smaller moment of inertia (*I*_A_) than those of symmetric mandibles.

Elastic energy is stored in the deformed shafts of both the symmetric snapping mandibles of *T. panamaensis* and the snap-jaw ant *M. camillae* (Fig. 1a)^5,6^, whereas energy storage in the asymmetric mandibles of *Pe. nitobei* is confined to the bendable ‘joint’ (or ‘pivot’ in Fig. 1c) at the curved part of the left mandible. The left mandible of a *Pe. nitobei* soldier is complex in shape, and this mandible is generally laterally compressed but wide along the dorsal–ventral axis (Fig. 1b,c). The location of minimum lateral thickness creates a ‘joint’ in the continuous and single-segmented mandible. The mandible base and its posterior part are more robust than its anterior counterpart is. Assuming homogeneous material composition, the anterior part of the mandible can rotate about the ‘joint’ by lateral pressure from the right mandible. The left mandible twists towards its tip to become laterally wide. Consequently, the left mandible tip becomes more difficult to bend and can effectively transfer impact energy for defence.

We suggest that because of its morphological modifications, the twisted left mandible of a *Pe. nitobei* soldier has a functional joint for storing elastic energy, which creates a shorter rotating section that requires less stored elastic energy to achieve greater velocity. In other words, asymmetric snapping mandibles are more efficient than symmetric snapping mandibles are.

### Antenna extension as an aiming behaviour

Because the direction of movement is unlikely to be corrected during high-speed ballistic movement, an aiming or triggering mechanism before initiating movement is crucial for precision^27,28^. For example, the archer fish *Toxotes jaculatrix* can identify the three-dimensional location of their insect target and precisely aim at and shoot it^28^. The snapping of a *Mystrium* snap-jaw ant’s mandible is triggered by sensory hairs on its labrum to ensure precise timing for catching rapidly moving prey^27^. The *Odontomachus* trap-jaw ant touches its prey using its elongated antennae before snapping, which may be a type of aiming behaviour^29^.

We observed that *Pe. nitobei* had a specific striking angle of 52.9° ± 22.7°, and it could hit active ants with a moderate probability of 8.6–34.0%. These results implied that *Pe. nitobei* aims before snapping to adjust its striking angle and distance from the target. We suggest that the forward extension of antennae observed in termite soldiers is an aiming behaviour and that the backward movement of the antennae beginning 45.9 ± 10.1 ms before snapping is a synergic behaviour (Fig. 2a). Because the snapping termite soldiers of *Pe. nitobei* lack eyes^30^, they likely locate enemies by touching with their antennae instead of by vision, as observed in *Odontomachus* trap-jaw ants^29^ and *T. panamaensis*^6^. This hypothesis was supported by the observation that the snapping behaviour of *Pe. nitobei* was triggered by touching their antennae tips with tweezers. However, forward-extended antennae may be at risk of being cut by the rapidly moving left mandible. Therefore, moving the antennae backward immediately before snapping can reduce this risk.

### Functional adaptation of asymmetric mandible snapping

The defensive strategies of termite soldiers are highly diversified and adapted to their habitats^10,31^. For example, *Cryptotermes* termite soldiers that live in single pieces of wood or dead tree branches use their heavy phragmotic heads to block enemies^12^. *Nasutitermes* termite soldiers, which construct arboreal nests and forage in trees, eject viscous secretions to defend the foraging party from ant attacks^32,33^. Biting or slashing mandibles are most commonly observed among primitive wood-feeding termites, such as most kalotermitids and rhinotermitids^34,35^.

Snapping mandibles have evolved in four independent clades of Termitidae and in one clade of Kalotermitidae^10,34,36,37^. These mandibles have been hypothesised to be a defensive weapon, especially in tunnels^6,12^, where both termites and their enemies are confined to narrow spaces (e.g., approximately 2-mm wide, as reported for *Pe. nitobei* and *Sinocapritermes mushae*^19,38^). Narrow tunnels limit termite movement as well as the gape of biting or slashing mandibles, thereby reducing their defensive performance. This study demonstrated that termites with asymmetric snapping mandibles strike at the front-left side without moving forward or opening their mandible wide. We suggest that asymmetric snapping mandibles are more advantageous than symmetric mandibles are when termites fight with intruding ants in tunnels.

**Figure 5:**
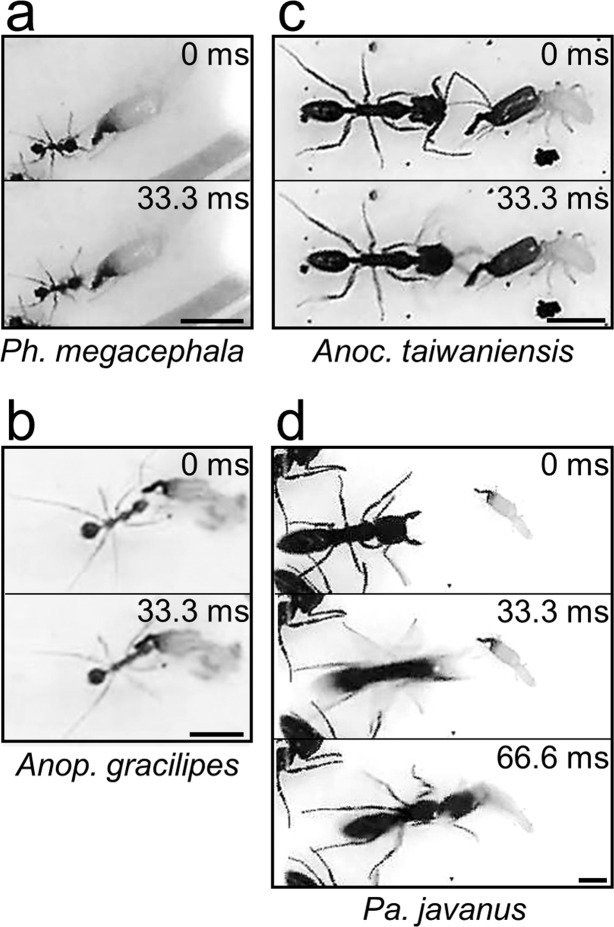
Attack behaviours of (**a**) *Pheidole megacephala*, (**b**) *Anoplolepis gracilipes*, (**c**) *Anoc. taiwaniensis*, and (**d**) *Pa. javanus* on *Pe. nitobei*. The attacks of *Ph*. *megacephala*, *Anop*. *gracilipes*, and *Anoc*. *taiwaniensis* were initiated after their antennae touched the mandibles or head of *Pe. nitobei* (0 ms), with a short distance between the heads of these three species and the mandible tip of *Pe. nitobei* (approximately 0.6–1.5 mm). By contrast, *Pa. javanus* initiated an attack when its head was approximately 6.8 mm away from the mandible tip of *Pe. nitobei* (0 ms). This attack was followed by rapid movement toward the termite’s head (33.3 ms) and clamping at its mandibles (66.6 ms).

The hypothesised function of termite mandibular snaps is to kill or push out ants^6,16^. We observed that the defensive function of *Pe. nitobei* mandibular snaps varied with the encountered ant species. Mandibular snaps could kill generalist predators but barely harmed specialist predators, particularly *Pa. javanus*, which is observed in most subterranean gallery systems of *Pe. nitobei* in the field (4/5 localities, unpublished data) and is likely the main predator of *Pe. nitobei*. *Pa*. *javanus* may be able to resist mandibular snaps for the following reasons: (1) *Pa*. *javanus* is larger than other ants (head width of approximately 2.5 mm for *Pa*. *javanus* vs. approximately 0.6–1.5 mm for other species); (2) the exoskeleton of *Pa. javanus* is thicker than that of most generalist and ponerine ants;^39^ and (3) *Pa. javanus* initiated long-distance attacking behaviour approximately 6.8 mm away from the mandible tip of *Pe. nitobei* (Fig. 5d), which was considerably longer than the attacking distances observed in *Ph. megacephala* (approximately 0.7 mm, Fig. 5a), *Anop. gracilipes* (approximately 0.6 mm, Fig. 5b), and *Anoc. taiwaniensis* (approximately 1.4 mm, Fig. 5c). Moreover, we observed that *Pa. javanus* could clamp the termite’s mandibles (Fig. 5d) to prevent counterattacks. Thus, we hypothesised that the aforementioned characteristics and behaviours of *Pa. javanus* were selected to withstand the stress of termites’ defensive behaviours, particularly powerful mandibular snaps. We also propose that the snapping mandible of *Pe. nitobei* can function as weapon to defend against their generalist but not specialist ant predators.

In conclusion, we provided evidence that the asymmetric snapping mandibles of *Pe. nitobei* are a rapid, powerful, and precise ballistic weapon. Asymmetric snapping behaviour was particularly advantageous in defending against intruding ants by striking them at their front side in subterranean tunnels.

## Supplementary information

Supplementary information.

Supplementary information2.

Supplementary information3.

Supplementary information4.

## Data Availability

Ultrahigh-speed video recordings are available as Supplementary Movies S1 and S2. Termite measurements are provided in the Supplementary Information. Video recordings and datasets of the ball-strike experiments and fights between termites and ants are available on request from the corresponding authors.

## References

[1] Patek SN (2015). The most powerful movements in biology. Am. Sci..

[2] Patek SN, Dudek DM, Rosario MV (2011). From bouncy legs to poisoned arrows: Elastic movements in invertebrates. J. Exp. Biol..

[3] Patek SN, Korff WL, Caldwell RL (2004). Deadly strike mechanism of a mantis shrimp. Nature.

[4] Brackenbury J, Hunt H (1993). Jumping in springtails: mechanism and dynamics. J. Zool., Lond..

[5] Larabee FJ, Smith AA, Suarez AV (2018). Snap-jaw morphology is specialized for high-speed power amplification in the Dracula ant, *Mystrium camillae*. R. Soc. Open Sci..

[6] Seid MA, Scheffrahn RH, Niven JE (2008). The rapid mandible strike of a termite soldier. Curr. Biol..

[7] Patek SN, Baio JE, Fisher BL, Suarez AV (2006). Multifunctionality and mechanical origins: Ballistic jaw propulsion in trap-jaw ants. Proc. Natl. Acad. Sci. USA.

[8] Tucker VA, Cade TJ, Tucker AE (1998). Diving speeds and angles of a gyrfalcon (*Falco rusticolus*). J. Exp. Biol..

[9] Nüchter T, Benoit M, Engel U, Özbek S, W HT (2006). Nanosecond-scale kinetics of nematocyst discharge. Curr. Biol..

[10] Scholtz OI, Macleod N, Eggleton P (2008). Termite soldier defence strategies: a reassessment of Prestwich’s classification and an examination of the evolution of defence morphology using extended eigenshape analyses of head morphology. Zool. J. Linnean Soc..

[11] Weesner, F. M. External anatomy. In Krishna, K. & Weesner, F. M. (eds.), *Biology of Termites* Vol. I (Academic Press, 1969).

[12] Deligne, J., Quennedey, A. & Blum, M. S. The enemies and defense mechanisms of termites. In Herman, H. R. (eds.), *Social Insects* Vol. II (Academic Press, 1981).

[13] Hölldobler, B. & Wilson, E. O. *The Ants*. (Springer-Verlag, 1990).

[14] Dejean A, Kenne M, Moreau CS (2007). Predatory abilities favour the success of the invasive ant *Pheidole megacephala* in an introduced area. J. Appl. Entomol..

[15] Fotso Kuate A, Tindo M, Hanna R, Kenne M, Goergen G (2008). Foraging activity and diet of the ant, *Anoplolepis tenella* Santschi (Hymenoptera: Formicidae), in southern Cameroon. African Entomol..

[16] Krishna K, Araujo RL (1968). A revision of the neotropical termite genus *Neocapritermes* (Isoptera, Termitidae, Termitinae). Bull. Am. Mus. Nat. Hist..

[17] Silvestri F (1902). Note preliminari sui Termitidi e Termitifili sudamericani. Boll. Mus. Turino.

[18] Silvestri F (1903). Contribuzione alla conoscenza dei termiti e termitofili dell’ America Meridionale. Portici. Redia.

[19] Chiu C-I, Yang M-M, Li H-F (2015). Structure and function of subterranean gallery systems of soil-feeding termites *Pericapritermes nitobei* and *Sinocapritermes mushae*. Ins. Soc..

[20] Leal IR, Oliveira PS (1995). Behavioral ecology of the neotropical termite-hunting ant *Pachycondyla* (= *Termitopone*) *marginata*: colony founding, group-raiding and migratory patterns. Behav. Ecol. Sociobiol..

[21] Schatz B, Orivel J, Lachaud JP, Beugnon G, Dejean A (1999). Sitemate recognition: the case of *Anochetus traegordhi* (Hymenoptera; Formicidae) preying on *Nasutitermes* (Isoptera: Termitidae). Sociobiology.

[22] Chiu C-I (2018). Foraging phenology of the fungus-growing termite *Odontotermes formosanus* (Blattodea: Termitidae). Environ. Entomol..

[23] Chiu C-I, Yeh H-T, Tsai M-J, Li H-F (2016). Naturalization and control of *Coptotermes gestroi* (Blattodea: Rhinotermitidae) in a Taiwanese forest. J. Econ. Entomol..

[24] Hewitt, P. G. *Conceptual Physics*. (Pearson Education Limited, 2014).

[25] R: A language and environment for statistical computing v. 3.0.2 (R Foundation for Statistical Computing, Vienna, Austria, 2013).

[26] Sharp N (1997). Timed running speed of a cheetah (*Acinonyx jubatus*). J. Zool..

[27] Gronenberg W (1996). The trap-jaw mechanism in the dacetine ants *Daceton armigerum* and *Strumigenys* sp. J. Exp. Biol..

[28] Schuster S, Wohi S, Griebsch M, Klostermeier I (2006). Animal cognition: How archer fish learn to down rapidly moving targets. Curr. Biol..

[29] Ehmer B, Gronenberg W (1997). Antennal muscles and fast antennal movements in ants. J. Comp. Physiol. B.

[30] Krishna K, Grimaldi DA, Krishna V, Engel MS (2013). Treatise on the Isoptera of the world. Bull. Am. Mus. Nat. Hist..

[31] Prestwich GD (1984). Defense-mechanisms of termites. Annu. Rev. Entomol..

[32] Eisner T, Kriston I, Aneshansley DJ (1976). Defensive behavior of a termite (*Nasutitermes exitiosus*). Behav. Ecol. Sociobiol..

[33] Prestwich GD (1979). Chemical defense by termite soldiers. J. Chem. Ecol..

[34] Krishna K (1961). A generic revision and phylogenetic study of the family Kalotermitidae (Isoptera). Bull. Am. Mus. Nat. Hist..

[35] Li H-F, Lan Y-C, Su N-Y (2011). Redescription of *Prorhinotermes japonicus* (Isoptera: Rhinotermitidae) from Taiwan. Ann. Entomol. Soc. Am..

[36] Inward DJ, Vogler AP, Eggleton P (2007). A comprehensive phylogenetic analysis of termites (Isoptera) illuminates key aspects of their evolutionary biology. Mol. Phylogenet. Evol..

[37] Scheffrahn RH, Bourguignon T, Akama DP, Sillam-Dusses D, Sobotnik J (2018). *Roisinitermes ebogoensis* gen. & sp. n., an outstanding drywood termite with snapping soldiers from Cameroon (Isoptera, Kalotermitidae). ZooKeys.

[38] Chiu C-I, Yang M-M, Li H-F (2016). Redescription of the soil-feeding termite *Sinocapritermes mushae* (Isoptera: Termitidae: Termitinae): The first step of genus revision. Ann. Entomol. Soc. Amer..

[39] Peeters C, Molet M, Lin C-C, Billen J (2017). Evolution of cheaper workers in ants: a comparative study of exoskeleton thickness. Biol. J. Linn. Soc..

