# Supplementary material for: Termite’s Twisted Mandible Presents Fast, Powerful, and Precise Strikes
Source: Sci Rep. 2020 Jun 11;10:9462. doi: 10.1038/s41598-020-66294-1 (PMC7289866; doi:10.1038/s41598-020-66294-1)
Supplement: Supplementary file 1 — Supplementary information. [file 41598_2020_66294_MOESM1_ESM.pdf]

Supporting Information

Title of paper:

Termite's Twisted Mandible Presents Fast, Powerful, and Precise Strikes

Kuan-Chih Kuan[1†], Chun-I Chiu[1†], Ming-Chih Shih[2], Kai-Jung Chi[2,3,4*], and Hou-Feng Li[1*]

[1]Department of Entomology, National Chung Hsing University, Taiwan
[2]Department of Physics and Institute of Biophysics, National Chung Hsing University, Taiwan
[3]Department of Life Sciences, National Chung Hsing University, Taiwan
[4]The iEGG and Animal Biotechnology Center, National Chung Hsing University, Taiwan
[†]Equal contribution
[*]Authors for correspondence:
Kai-Jung Chi, email: kjchi@phys.nchu.edu.tw
Hou-Feng Li, email: houfeng@nchu.edu.tw

This document includes:

Tables S1-S3
Legends for Supplementary Movies S1-S3

Table S1. Collection localities and the sample size used in different experiments

Experiment	Sample size	Collection locality
Observing snapping behavior using high-speed video camera (1,000 fps)	10	Dakeng, Taichung
Recording the motion of mandibles using ultrahigh-speed video camera (460,830 fps) [1]	2/5	Dakeng, Taichung
Recording the motion of metal balls	6	Xiaping Tropical Botanical Garden, Nantou
using high-speed video camera	7	Huisun Forest Station, Nantou
(1,000 fps)	2	Dakeng, Taichung
Measuring the size of left mandible	17	Xiaping Tropical Botanical Garden, Nantou
1. Mandible length	17/17	
2. Mandible mass[2]	14/17	
Observing the fights between termites and ants[3]		
2019/07/20, 4 replications[4] were conducted at the same time	21	Huisun Forest Station, Nantou
1. Fight with *P. megacephala*	16/21	
2. Fight with *Anop. gracilipes*[5]	16/21	
3. Fight with *Anoc. taiwaniensis*[6]	16/16	
4. Fight with *E. javanus*	0/5	
2019/09/01, 2 replications[4] were conducted at the same time	32	Huisun Forest Station, Nantou
1. Fight with *P. megacephala*	8/32	
2. Fight with *Anop. gracilipes*	8/32	
3. Fight with *Anoc. taiwaniensis*[7]	8/32	
4. Fight with *E. javanus*[8]	24/30	
Total number of termites collected	100	

[1] three termites died before performing snapping behavior;

[2] three mandibles were broken during dissection;

[3] *P. megacephala* and *Anop. gracilipes* were collected from National Chung Hsing University, Taichung. *Anoc. taiwaniensis* and *E. javanus* were collected from Sun Moon Lake, Nantou. The fighting experiments were conducted in order. The ants used in all replications were different individuals. Termites were not repeatedly used to fight with both *Anoc. taiwaniensis* and *Pa. javanus*;

[4] four termites and four ants were used in each replication;

[5] five termites died in the fights with *Anop. gracilipes*;

[6] 11 termites died in the fights with *Anoc. taiwaniensis*;

[7] two termites died in the fights with *Anoc. taiwaniensis*;

[8] 15 termites died in the fights with *E. javanus*.

Table S2. Body size of termites and the results of ball-strike experiments

Locality	ID#	n	M_T (mg)	L_T (mm)	V_B (m/s)	V_T (m/s)	ω_T (rad/s)
Dakeng	A	7	3.26	5.9	0.85±0.41	0.58±0.14	279.0±265.1
	B	8	3.39	6.0	1.51±0.45	1.12±0.47	1105.5±297.9
	C	7	2.91	5.9	1.20±0.37	0.98±0.11	1026.4±181.6
	D	5	3.13	6.5	1.29±0.27	1.29±0.51	904.6±93.1
	E	7	3.09	6.3	1.63±0.48	1.22±0.51	1046.7±302.1
	F	5	3.31	7.0	1.83±0.69	1.38±0.43	924.6±307.6
Huisun Forest Station	G	11	3.39	6.1	1.69±0.64	1.25±0.50	1002.2±273.5
	H	14	3.14	6.1	1.33±0.42	1.08±0.36	924.2±399.7
	I	3	3.72	6.1	1.09±0.62	0.76±0.25	1207.7±627.5
	J	3	3.81	6.3	1.63±0.39	0.79±0.44	860.6±344.2
	K	5	3.15	5.8	1.23±0.38	0.80±0.32	919.1±238.9
	L	5	3.50	5.9	1.75±0.46	1.07±0.32	1030.7±240.0
	M	3	3.09	5.5	1.65±0.08	1.26±0.17	1116.4±120.9
Xiaping Tropical Botanical Garden	N	6	2.91	6.2	0.91±0.31	0.90±0.14	604.9±370.8
	O	3	3.2	6.1	1.40±0.25	1.11±0.08	942.0±271.9

n: number of trials

M_T, L_T: body mass and length of the soldier termites.

V_B, V_T, ω_T: linear velocities of the ball and termite, and the angular velocity of the termite after ball-strike.

Table S3. Mass and length of left mandibles from *P. nitobei* soldier termites.

Mandible section	Length, L (mm)		Mass, M (mg)	
	mean±SD	n	mean[a]	n
Anterior part	L_A: 1.056±0.045	17	A_1: 0.008<br>A_2: 0.020	12/14
Posterior part	0.922±0.027	17	0.056	14/14

Definitions of L_A, A_1, A_2, and the posterior part of mandible, see Figs. 1b and 1c.

[a] the masses of anterior and posterior parts were estimated as the means of 12 and 14 mandible parts, respectively, to reduce the error due to their small size.

Legends for Supplementary Movies

Movie S1. Single snap of a *Pericapritermes nitobei* soldier recorded by an ultrahigh-speed video camera at 460,830 fps. As shown in Fig. 2b, this mandibular snap was performed over 21.7 μs. The peak linear velocity at 8.68 μs after execution was 132.4 m/s. This video was scaled up and sharpened by using FFmpeg (v. 4.2.2), and the text was added by using Python (v. 3.8) with the package *PIL*.

Movie S2. Single snap of a *Pericapritermes nitobei* soldier recorded by an ultrahigh-speed video camera at 460,830 fps. As shown in Fig. 2c, this mandibular snap was performed over 43.4 μs. The peak linear velocity at 8.68 μs after execution was 89.7 m/s. This video was scaled up and sharpened by using FFmpeg (v. 4.2.2), and the text was added by using Python (v. 3.8) with the package *PIL*.

Movie S3. Motion of the termite soldier and metal ball in a ball-strike experiment recorded by a high-speed video camera at 1,000 fps. After a single snap, the termite soldier and metal ball moved away from each other. Analyses of these movements allowed us to estimate the snapping velocity, force, and accuracy (as shown in Fig. 3). The text was added by using Python (v. 3.8) with the package *PIL*.
